# Establishment of the monomeric yellow-green fluorescent protein mNeonGreen for life cell imaging in mycelial fungi

**DOI:** 10.1186/s13568-020-01160-x

**Published:** 2020-12-21

**Authors:** Antonia Werner, Kolja L. Otte, Gertrud Stahlhut, Stefanie Pöggeler

**Affiliations:** Department of Genetics of Eukaryotic Microorganisms, Institute of Microbiology and Genetics, Georg-August-University of Göttingen, Grisebachstr. 8, 37077 Göttingen, Germany

**Keywords:** mNeonGreen, *Sordaria macrospora*, Peroxisomes, Fluorescent protein

## Abstract

The engineered monomeric version of the lancelet *Branchiostoma lanceolatum* fluorescent protein, mNeonGreen (mNG), has several positive characteristics, such as a very bright fluorescence, high photostability and fast maturation. These features make it a good candidate for the utilization as fluorescent tool for cell biology and biochemical applications in filamentous fungi. We report the generation of plasmids for the expression of the heterologous mNG gene under the control of an inducible and a constitutive promoter in the filamentous ascomycete *Sordaria macrospora* and display a stable expression of mNG in the cytoplasm. To demonstrate its usefulness for labeling of organelles, the peroxisomal targeting sequence serine-lysine-leucine (SKL) was fused to mNG. Expression of this tagged version led to protein import of mNG into peroxisomes and their bright fluorescence in life cell imaging.

## Introduction

Fluorescence microscopy is a potent method for studying in vivo protein localization, cytoskeleton dynamics, infections and protein–protein-interactions in filamentous fungi (Berepiki et al. 2010; Chapuis et al. 2019; Hoff and Kück 2005; Lorang et al. 2001).

Fluorescent proteins can be expressed in fungal cells by inserting genes encoding fluorescent proteins into the genome under the control of constitutive or inducible promoters (Oda et al. 2016; Pöggeler et al. 2003). The green fluorescent protein GFP from the jellyfish *Aequorea victoria* and its engineered versions with altered spectral properties, such as yellow and cyan-shifted mutants, enhanced brightness versions or pH-sensitive variants have been utilized in fungal cell biology for many years (Bagar et al. 2009; Chalfie et al. 1994; Chudakov et al. 2005; Lorang et al. 2001; Rech et al. 2007).

In addition, the tetrameric fluorescent protein DsRed, its monomeric variant mRFP from the marine anemone *Discosoma striata* as well as brighter versions of mRFP (mCherry and tdTomato) emitting in the red light spectrum have been successfully expressed in filamentous fungi (Campbell et al. 2002; Mikkelsen et al. 2003; Schuster et al. 2015; Toews et al. 2004; Xiao et al. 2018). Furthermore, the red fluorescent TagRFP-T, from the sea anemone *Entacmaea quadricolor* has been applied in fungal life cell imaging (Schuster et al. 2015).

Recently, the new and very bright yellow fluorescent protein LanYFP with a high quantum yield (~ 0.95) and extinction coefficient (~ 150,000 M − 1 cm^−1^) was isolated from lancelet *Branchiostoma lanceolatum*. Engineering of the tetrameric LanYFP resulted in a 26.5 kDa monomeric, yellow–green fluorescent protein called mNeonGreen (mNG) (Shaner et al. 2013). In comparison to LanYFP, mNG harbors 21 substitutions and contains modified N- and C-termini of additional 11 and 7 amino acids derived from eGFP. mNG has a blue-shifted excitation maximum of 506 nm and an emission maximum of 517 nm with a high quantum yield excitation coefficient. Compared to eGFP it has a nearly threefold brighter emission, an increased photostability and shorter maturation time (Shaner et al. 2013). The new yellow- green fluorescent protein has been used mainly for fluorescent microscopy. It has been applied as genetically encoded tag to visualize gene products and cellular compartments in bacteria, animals, plants and yeast (Botman et al. 2019; Heppert et al. 2016; Hostettler et al. 2017; Stoddard and Rolland 2019; Wilton et al. 2017). Only one report exists on expression mNG in filamentous fungi. Recently, in *Aspergillus fumigatus* a version of mNG codon-optimized for the green algae *Chlamydomonas rheinhardii* was shown to be expressed under control of the strong constitutive *tefA* promoter (Pham et al. 2020).

Often expression of fluorescent proteins in filamentous fungi requires species-specific variants that can be efficiently transcribed and translated in the respected host. Therefore, often codon-optimization of the coding region and a fungal promoter that meets the conditions for a specific experimental set-ups have to be established (Lorang et al. 2001).

In this study, we demonstrate the successful expression of mNG in the filamentous ascomycete *Sordaria macrospora*, a model organism for investigating fruiting body development and meiosis in fungi (Teichert et al. 2014, 2020; Zickler and Espagne, 2016). We generated plasmids for expression of *mng* under the control of the constitutive *gpd* promoter of *Aspergillus nidulans* and the xylose inducible *Smxyl* promoter of *S. macrospora* (Bloemendal et al. 2014). To demonstrate the usefulness of mNG for visualization of fungal organelles, we fused the peroxisomal localization signal SKL to mNG and monitored peroxisomes in *S. macrospora*.

## Material and methods

### Strains, media and growth conditions

*Escherichia coli* strain MACH1 (Thermo Fisher Scientific, C862003, Waltham, USA) was used for cloning and propagation of recombinant plasmids using standard culture conditions (Sambrook et al. 2001). Homologous recombination was performed to generate recombinant plasmids in *Saccharomyces cerevisiae* strain PJ69-4A (Colot et al. 2006; James et al. 1996). *S. macrospora* strains were transformed with recombinant plasmids as described previously (Walz and Kück 1995). Selection of transformants was performed on media containing nourseothricin-dihydrogen sulphate (50 µg ml^−1^) (Jena Bioscience GmBH, AB-102XL, Jena, Germany). *S. macrospora* strains were grown at 27 °C on liquid or solid biomalt maize medium (BMM), or *Sordaria* Westergaard (SWG) fructification medium under continuous light conditions (Elleuche and Pöggeler 2008; Esser 1982; Nowrousian et al. 2005). To induce the xylose promoter, SWG medium was prepared with 2% xylose replacing glucose.

### Generation of plasmids

All primers and template plasmids used for PCR amplifications are listed in Tables 1 and 2, respectively. Primers were synthesized by Sigma-Aldrich Chemie GmbH (Taufkirchen, Germany).

**Table 1:** List of primers used in this study

Name	Sequence (5′–3′)
Pxyl_fw	^**GACTGGTCTCA**^^**AGTC**^GAACCTTCTCTTCTCCATTTCTT
Pxyl_rev	^**CAGAGGTCTCA**^^**GCAG**^GTTGGCGGTTTCTGGTTAGGCC
Neon_fw	^**CAGAGGTCTCA**^^**CTGC**^ATGGTGAGCAAGGGCGAGGAGG
Neon_rev	^**CTCAGGTCTCC**^^**CGTA**^CTACTTGTACAGCTCGTCCATGC
Pxylneon_fw	^**GTAACGCCAGGGTTTTCCCAGTCACGACG**^GAACCTTCTCTTCTCCATTT
Pxylneon_rev	^**GCGGATAACAATTTCACACAGGAAACAGC**^CTACTTGTACAGCTCGTCCA
trpC-neon	^**GCCTTTACCGATGTGATGGGCATGGACGAGCTTTACAAGTAG**^AGCGGCCGCTCTAGAACTAGTGGATCCACT
TtrpC_pRS_r	^**GCGGATAACAATTTCACACAGGAAACAGC**^TCGAGTGGAGATGTGGAGTGG
Neon_fw-2	^**CGAGTCAGGTCTCC**^^**TGGT**^ATGGTGAGCAAGGGCGAG GAGG
Neon_rev-2	^**ACCGACAGGTCTCG**^^**ATCC**^CTTGTACAGCTCGTCCATGC
Neo_f	ATGGTGAGCAAGGGCGAG
Neo_SKL_TrpC_r	^**GTTTGATGATTTCAGTAACGTTAAGTGGA**^***TTAGAGCTTGCT***CTTGTACAGCTCGTCCATGC
pRS_Pgpd_f	^**GTAACGCCAGGGTTTTCCCAGTCACGACG**^GTACAGTGACCGGTGACTCT
Pgpd_Neo_r	^**ATGTTATCCTCCTCGCCCTTGCTCACCAT**^GGGAAAAGAAAGAGAAAAGAAAAGAGCA
TtrpC_f	TCCACTTAACGTTACTGAAATCATCAAAC

bold superscript = overhang; superscript underlined = the four base pairs after the *Bsa*I recognition site in the overhang, bold italics = tripeptide SKL and a stop codon

*T* terminator, *P* promotor

**Table 2:** List of plasmids used in this study

Plasmid	Characteristics	Source
pRS-nat	*amp*^*R*^*, ura3, nat*^*R*^	(Klix et al. 2010)
p1783-1	*amp*^*R*^*, ura3, hyg*^*R,*^ *Pgpd::egfp::TtrpC*	(Pöggeler et al. 2003)
pRHN1	*amp*^*R*^*, ura3, nat*^*R*^ *Pgpd::Dsred::Ttrpc*	(Janus et al. 2007)
pDsRed-SKL	*amp*^*R*^*, nat*^*R*^ *Pgpd::Dsred-SKL::TtrpC*	(Elleuche and Pöggeler 2008)
pDest::amp	*amp*^*R*^	Dahlmann et al. 2020
pGG-C-F	*amp*^*R*^*,, nat*^*R*^ *Pgpd::3xFLAG::TtrpC*	Dahlmann et al. 2020
pNpX-GFP	*amp*^*R*^*, ura3, nat*^*R*^ *Pgpd::egfp::TtrpC*	(Bloemendal et al. 2014)
753-pENTR20_mNeonGreen-N1	*kan*^*R*^*, mng (optimized)*	Schink, pers. communication
pxyl-mNeonGreen	*amp*^*R*^*, ura3, nat*^*R*^ *Pxyl::mng*	This study
pxyl-mng	*amp*^*R*^*, ura3, nat*^*R*^ *Pxyl::mng::TtrpC*	This study
pGG-C-F-mng	*amp*^*R*^*, nat*^*R*^ *Pgpd::mng::3xFLAG::TtrpC*	This study
pmng-SKL	*amp*^*R*^*, ura3, nat*^*R*^ *Pgpd::mng-SKL::TtrpC*	This study

*nat*^R^: nourseothricin resistant, *hyg*^*R*^: hygromycin resistant; *amp*^R^: ampicillin resistance; *ura3,* Orotidine-5′-phosphate decarboxylase gene of *S. cerevisiae; Pxyl:* promoter of the xylose gene *Smxyl* of *Sordaria macrospora; Pgpd:* promoter of the glycerinaldehyd-3-phosphat-dehydrogenase-gene of *Aspergillus nidulans; TtrpC:* terminator of the anthranilat synthase gene of *Aspergillus nidulans*; ssi: single spore isolate; SKL: peroxisomal targeting sequence Ser-Arg-Leu; *mng*: gene for green fluorescence protein monomeric NeonGreen, (mNG) of *Branchiostoma lanceolatum*; *Dsred*: gene for red fluorescence protein (DsRed) of *Discosoma* species; *egfp*: gene for green fluorescence protein enhanced green fluorescent protein (eGFP) of *Aequorea victoria*

**Fig. 1:**
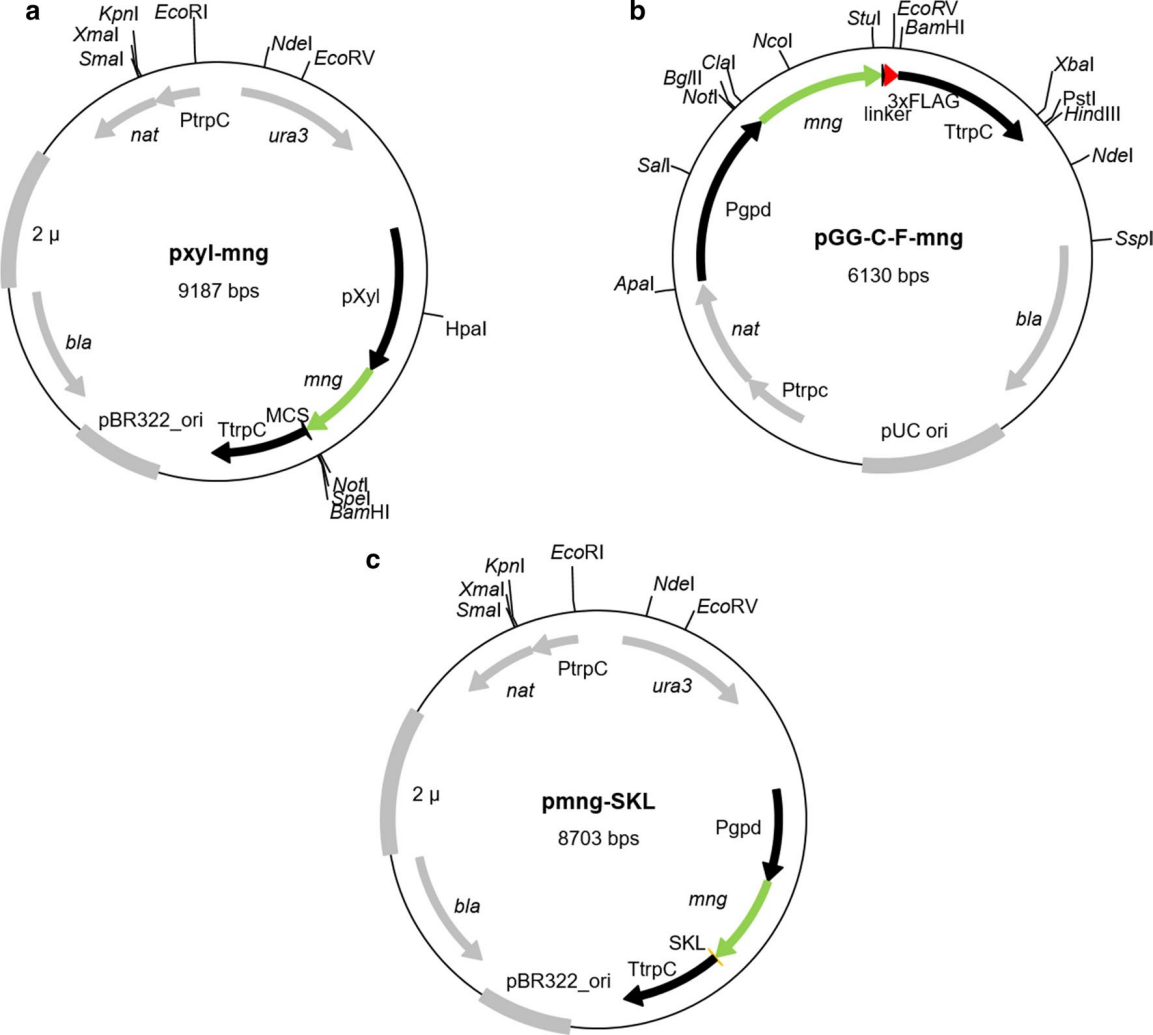
Physical maps of mNG plasmids. **a** pxyl-mng, **b** pGG-C-F-mng, **c** pmng-SKL The ampicillin resistance cassette (*bla*) and the ori for selection and replication in *E. coli* are indicated. The nourseothricin resistance gene *nat1* is under control of the *A. nidulans trpC* promoter (PtrpC) can be used for selection of fungal transformants. The 2 µ ori and the *ura3* gene can be used for transformation of *S. cerevisiae*. The *mng* ORF (green arrow) is either regulated by the xylose inducible *Smxyl* promoter of *S. macrospora* (Pxyl) or the *gpd* promoter *A. nidulans* (Pgpd) and the *trpC* terminator of *A. nidulans* (TtrpC). Plasmid pxyl-mng contains a multiple cloning site (MCS). In plasmid pGG-C-F-mng a C-terminal 3 × FLAG tag: (DYKDHD-G-DYKDHD-I-DYKDDDDK, red arrow) is fused via a short linker (GSGSG) to mNG. In plasmid pmng-SKL the peroxisomal targeting sequences SKL (orange arrow) is fused to the C-terminus of mNG

To generate a plasmid in which mNG is under control of an inducible promoter we used the Golden Gate cloning system (Terfrüchte et al. 2014). This system uses restriction enzyme *Bsa*I hydrolyzing 4 base pairs after its recognition site, independent from the identity of these four base pairs, which allows the generation of sticky overhangs, which can be used for complex fragment assemblies (Dahlmann et al. 2020). For the PCR amplification of the *S. macrospora xyl* promoter *Smxyl*, plasmid pNpX-GFP (Bloemendal et al. 2014) was used as template and Pxyl_fw and Pxyl_rev as primers. To amplify the gene coding for mNG, plasmid 753-pENTR20_mNeonGreen-N1 (a kind gift from Kay Oliver Schink, Centre for Cancer Biomedicine (CCB) Oslo University Hospital, Radiumhospitalet) served as template for PCR with primer pair Neon_fw/Neon_rev. The *Smxyl*-promoter fragment and the *mng* fragment were combined with vector pDest::amp (Dahlmann et al. 2020) by treatment with *Bsa*I and T4 DNA-ligase. The resulting vector was used to amplify the *Pxyl-mng* fragment with the primer pair Pxylneon_fw/Pxylneon_rev. Both primers generated a 29-bp overhang at the PCR product which was together with *Xho*I linearized vector pRS-nat used for homologous recombination cloning in *Saccharomyces cerevisiae* strain PJ69-4A (Colot et al. 2006). Since the resulting plasmid pxyl-mNeonGreen revealed no fluorescence after transformation into *S. macrospora*, we decided to introduce the terminator sequence of the *trpC* gene of *Aspergillus nidulans* downstream to the *mng* coding sequence. Plasmid pxyl-mNeonGreen was linearized with *BsRG*I and the *A. nidulans trpC* terminator was amplified with primer pair trpC-neon/TtrpC_pRS_r. Homologous recombination in *S. cerevisiae* resulted in plasmid pxyl-mng (Fig. 1a). To express mNG under the constitutive *gpd* promoter of *A. nidulans*, we utilized the Golden Gate cloning vector pGG-C-F. This vector contains a 3 × FLAG tag flanked by the *gpd* promoter and the *trpC* terminator of *A. nidulans* (Teichert et. al. unpublished). The *mng* ORF without STOP codon was PCR amplified with the primer pair Neon_fw-2/Neon_rev-2 and 753-pENTR20_mNeonGreen-N1 served as template. The resulting PCR fragment was combined with vector pGG-C-F. Incubation with *Bsa*I and T4 DNA-ligase resulted in plasmid pGG-C-F-mng (Fig. 1b).

To label peroxisomes with mNG, we fused the peroxisomal targeting sequence 1 (PTS1) encoding the three amino acids serine-lysine-leucine (SKL) to the C-terminus of the *mng* ORF. The coding sequence of the *mng* gene was amplified from plasmid 753_pENTR20_mng-N1 with primer pair Neo_f/Neo_SKL_TrpC_r. The base-triplets encoding the SKL-motif is contained in the overhang of primer Neo_SKL_TrpC_r and was adapted to the codon usage of *S. macrospora* according to the HIVE-Codon Usage Table (https://hive.biochemistry.gwu.edu/review/codon2) (Athey et al. 2017). To put the *mng-SKL* gene under control of the constitutive *gpd* promoter and *trpC* terminator of *A. nidulans*, we amplified the promoter and terminator from plasmid p1783-1 with primer pair pRS_Pgpd_f/Pgpd_Neo_r and TtrpC_f /TtrpC_pRS_r, respectively. The three PCR products were cloned into *Xho*I-linearized vector pRS-nat by homologous recombination in yeast strain PJ69-4A (Colot et al. 2006). The resulting plasmid was designated pmng-SKL (Fig. 1c). DNA sequencing of the plasmids was performed by Seqlab Sequence Service Laboratories GmbH (Göttingen, Germany).

### Generation of *S. macrospora* strains

All strains used in this study are listed in Additional file 1: Table S1. Strains were generated by transforming the plasmids (pxyl-mng, pGG-C-F-mng, pmng-SKL) presented in Table 2 into the *S. macrospora* wild type strain. Strains with ectopically integrated fusion constructs were selected on BMM medium supplemented with nourseothricin-dihydrogen sulphate (Hoff and Kück 2006; Walz and Kück 1995). Primary transformants were crossed with the color spore mutant fus1-1 (Nowrousian et al. 2012) to obtain single spore isolates (ssi) as described previously (Bernhards and Pöggeler, 2011). At least two independent ssi were analyzed in fluorescent microscopy.

### Protein sample preparation and Western-blot analysis

*S. macrospora* strains were cultivated in corresponding media for three days. For the protein preparation, mycelium was harvested, dried, and ground in liquid nitrogen. 1 g of mycelium was combined with 560 µl extraction buffer (100 mM Tris–HCl pH 7.5, 150 mM NaCl, 2 mM EDTA) with freshly added 2 mM DTT, 1 mM PMSF, 0.2% NP-40, protease inhibitor cocktail IV (1tbl/50 ml, 04693132001, Mannheim, Germany) and PhosSTOP™ (1 tbl/10 ml, Roche, 04906837001, Mannheim, Germany). Protein samples were centrifuged for 20 min at 12,000 rpm and 4 °C. Afterwards, 45 µl of the supernatant was boiled with 50 µl of 2 × Tris-glycerine/ SDS-sample buffer (Serva, P190078, Heidelberg, Germany) and 5 µl of 1 M DTT for 2 min at 85 °C. 15 µl of the samples were loaded on a 12% SDS gel, together with the Nippon Genetics Co. Europe blue star pre-stained protein marker (NIPPON Genetics Europe, MWP03, Düren, Germany). Proteins separated in SDS-PAGE were transferred to a AmershamTM ProtranTM Nitrocellulose Blotting Membrane (GE Healthcare, RPN203B, Little Chalfont, UK) applying 300 mA a semi-dry device (Biometra, Fastblot 014–200 type B34, Göttingen, Germany) (Towbin et al. 1979). Transfer of proteins to the membrane was visualized by staining the membrane with Ponceau red solution (3% TCA, 0.2% Ponceau S) for 45 min and washing with A.dest.

Nitrocellulose membranes containing transferred proteins were washed once with 1 × Tris-buffered saline supplemented with 0.05% Tween 20® (TBST) for 15 min and blocked with 5% (w/v) milk powder in TBST for 1 h at room temperature. Antigen–antibody reaction was performed using a mNeonGreen-antibody (1:5000; ChromoTek GmbH, 32f6-20, Planegg-Martinsried, Germany) and a horse-radish peroxidase (HRP) coupled secondary anti mouse-antibody (1:10,000 Sigma-Aldrich, A5278) solved in 5% skim milk/TBST. The membrane and the primary antibody solution were incubated over night at 4 °C. After the antibody was removed, the membrane was washed three times for 15 min with TBST. The secondary antibody was applied to the membrane for 1 h at room temperature and the membrane was washed three times with TBST. Enhanced chemiluminescent reaction was used to detect the HRP coupled antibodies using the ImmobilonTM Western HRP Substrate kit (Merck, WBKLS0500, Kenilworth, New Jersey, USA).

### Microscopy

To ensure that fungal cultures of identical developmental stages were compared in light and fluorescence microscopy, strains were grown for 24 h at 27 °C on BMM or SWG medium with cellophane and investigated with the AxioImager M1 microscope (Zeiss, Jena, Germany) (Rech et al. 2007). Microspcopic analysis was performed with at least two independent transformats in at least three replicates. Images were captured using a Photometrix CoolSNAP HQ camera (Roper Scientific, Photometrics, Tucson, AZ, USA) and image processing was done using ZEISS ZEN Digital Imaging (version 2.3; Zeiss): Exposure time was identical for all images. For the detection of the eGFP and mNG signals Chroma filter set 49002 (exciter ET470/40x, ET525/50 m, beamsplitter T495lpxr) and for DsRed Chroma filter set 49005 (exciter ET545/30x, emitter ET620/60 m and beamsplitter T570LP) were used. In addition, we used YFP Chroma filter set 49003 (exciter ET500/20x, emitter ET535/30 m and beamsplitter T515LP) for fluorescent mNG signals.

## Results

### Construction of fungal mNeonGreen reporter plasmids

The visualization of fluorescent reporter proteins is an important technique in fungal cell biology. Here, we examined the utilization of the lancelet yellow-green fluorescent protein for fluorescent microscopy in the filamentous ascomycete *S. macrospora*. From our experience, codon-usage optimized human genes encoding for fluorescent proteins perform best in *S. macrospora* (Pöggeler et al. 2003). Therefore, we employed a human codon-optimized version of the mNG gene, kindly provided by Dr. Kay Oliver Schink (Centre for Cancer Biomedicine (CCB) Oslo University Hospital, Radiumhospitalet). This *mng* version harbors 35 silent base mutations to be human codon-optimized for high expression levels in human cells (Additional file 1: Figure S1). In comparison to the original sequence [KC295282.1, (Shaner et al. 2013)], none of the four least used codons of *S. macrospora* are present in this human codon-optimized *mng* gene (Athey et al. 2017). Furthermore, with a 55.84% GC-content the humanized *mng* version is very similar to the 56.5% GC-content of coding regions in *S. macrospora* (Nowrousian et al. 2012).

To avoid a putative toxicity of mNG we first cloned *mng* under control of the xylose inducible promoter *Smxyl* of *S. macrospora* in plasmid pxyl-mng (Bloemendal et al. 2014) (Fig. 1a). The vector contains three unique sites of the commonly used restriction enzymes *Not*I, *Spe*I, and *Bam*HI. All sites are located downstream of the *mng* gene and can be employed to C-terminally fuse fungal genes of interest. In plasmid pGG-C-F-mng, *mng* is under control of the constitutive *A. nidulans gpd* promoter and is further fused to a 3 × FLAG Tag, which can be used for Western-blot experiments with a commercially available FLAG antibody (Fig. 1b). Further, we constructed a mNG-SKL plasmid for labelling of peroxisomes, carrying *mng-SKL* under control of the constitutive *gpd* promoter as well (Fig. 1c).

### Expression of mNG in *S. macrospora*

**Fig. 2:**
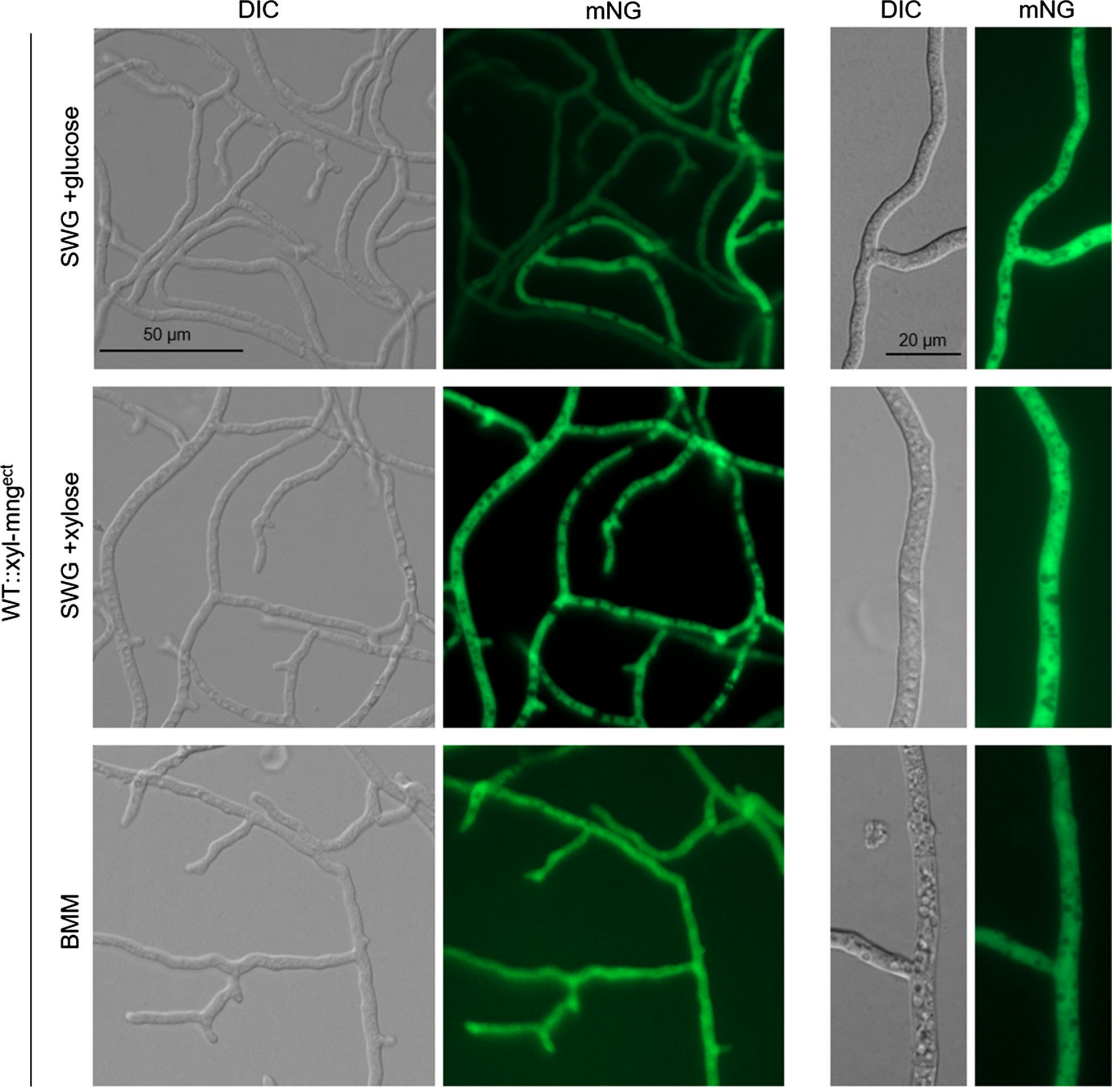
Fluorescence microscopy to demonstrate *mng* expression under control of the *Smxyl* promoter, grown under non-inducing (SWG + glucose, BMM) and inducing conditions (SWG + xylose). The exposure time for image acquisition under both conditions was set to be the same. Scale bar, 50 and 20 µm

First, we applied plasmid pxyl-mng for transformation of *S. macrospora* wild type. Since this plasmid encodes mNG under control of the xylose inducible *Smxyl* promoter, we tested expression of the *mng* gene under different growth conditions by means of fluorescence microscopy and Western-blot analysis. Cultures were grown in synthetic Westergaards (SWG) medium (Nowrousian et al. 2005) supplemented with either glucose or xylose as the sole carbon source or on complex corn meal medium (BMM) (Towbin et al. 1979). In fluorescence microscopy, under all growth conditions the yellow green fluorescence signal was detectable in the cytoplasm of the transformants. The signal was clearly excluded from the vacuoles. The strongest signal could be observed under inducing conditions (SWG + xylose), whereas a weaker signal was detectable under repressed conditions (SWG + glucose) and growth in the complex BMM medium (Fig. 2).

**Fig. 3:**
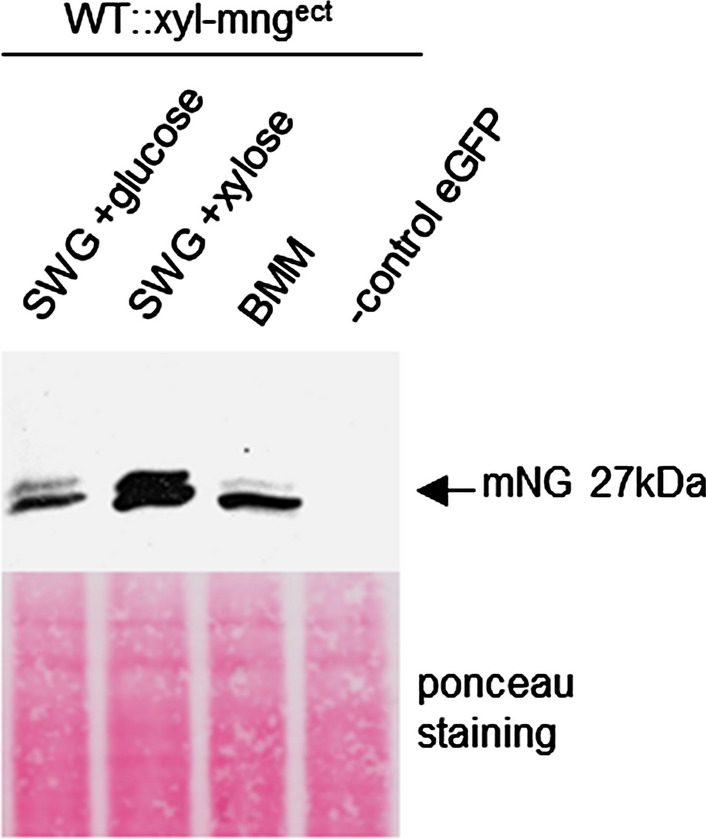
Western-blot analysis of total protein extracts to demonstrate *mng* expression under control of the *Smxyl* promoter grown under non-inducing (SWG + glucose) and inducing conditions (SWG + xylose), as well as in BMM. An *S. macrospora* wt transformant carrying plasmid p1783 and expressing free eGFP served as a control. Ponceau red staining of the blot before the immunodetection served as loading control

These results were verified by Western-blot analysis (Fig. 3). After growth for 3 days in the respected liquid medium, mycelium was harvested and proteins were extracted from the transformants for SDS–PAGE and Western-blot analysis with an anti-mNeonGreen-antibody. We used transformants expressing eGFP as a negative control. A clear mNG signal of around 27 kDa was detectable for all three conditions. The strongest signal was visible under inducing conditions (SWG + xylose). In all lanes, a second band at slightly higher molecular weight than expected can be observed. This may be due to glycosylation or other protein modifications. mNG has only very little sequence identity in common with eGFP (Stoddard and Rolland 2019), therefore no cross reactivity of the mNeonGreen antibody was visible with eGFP (Fig. 3). These data indicate that the human codon optimized version of the *mng* gene can be expressed under control the inducible *Smxyl* promoter.

**Fig. 4:**
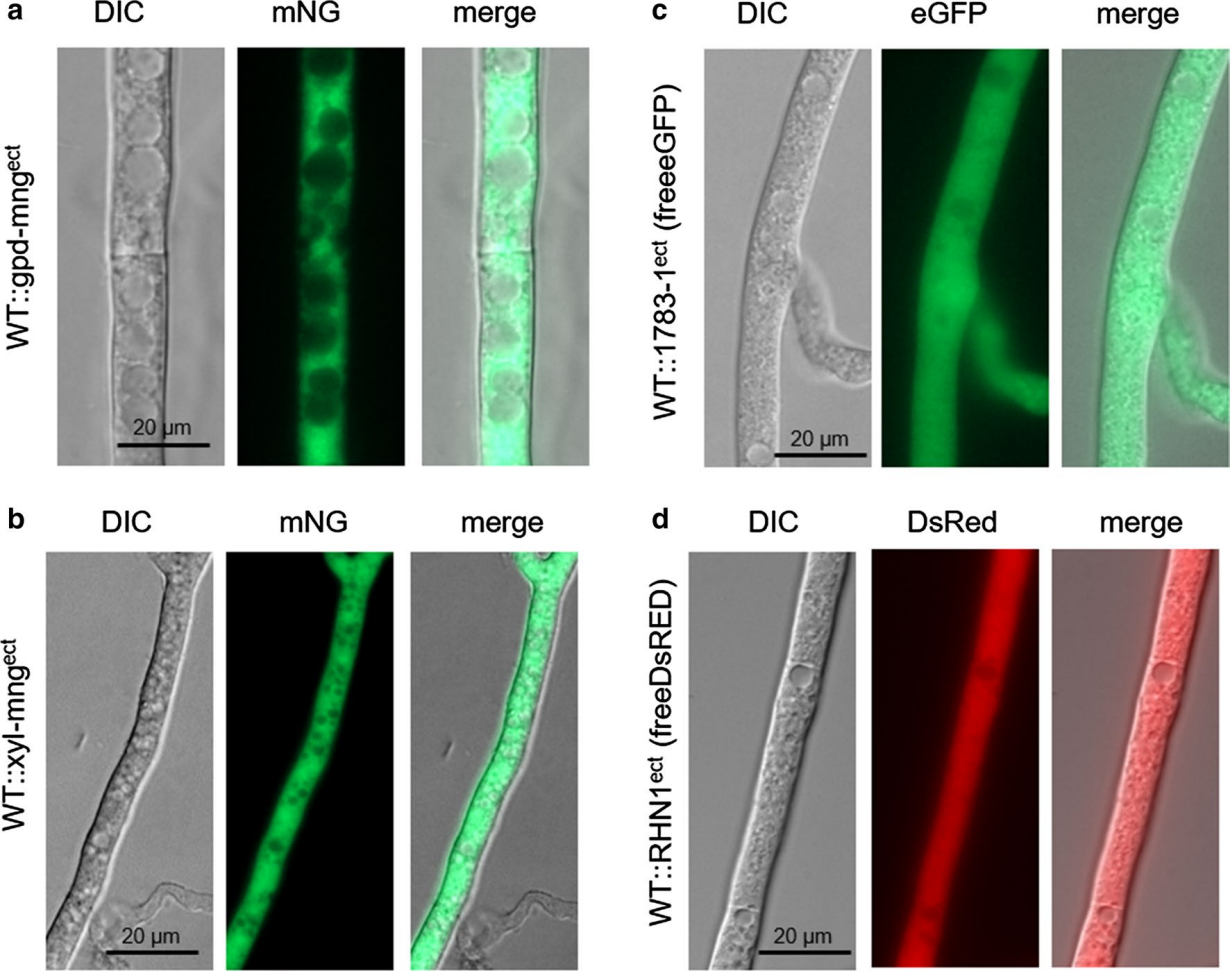
Fluorescence microscopy of cytosolic-targeted mNG, eGFP and DsRed in *S. macrospora*. **a** *mng* expression under control of the constitutive *gpd* promoter of *A. nidulans*, **b** *mng* expression under control of the induced *Smxyl* promoter, **c** expression of *egfp* under control of the *gpd* promoter, **d** expression of DsRed under the control of the *gpd* promoter. The exposure time for image acquisition of all strains was set to be the same. Scale bar, 20 µm

Next, we tested the expression of the *mng* gene under control of the strong constitutive *gpd* promoter of *A. nidulans.* Western-blot analysis with the mNeonGreen antibody revealed a strong signal (Additional file 1: Figure S2). Compared to strains expressing DsRed or eGFP under control of the *gpd* promoter the fluorescence signal of mNG appeared almost equal (Fig. 4). The fluorescence intensity of strains expressing *mng* under control of the constitutive *gpd* promoter is similar to strains expressing *mng* under control of the inducible *Smxyl* promoter upon growth under inducing conditions (Fig. 4a, b). In all strains, the fluorescence was uniformly visible throughout the cytoplasm of the hyphae and appeared to be excluded from the vacuoles.

In comparison to EGFP (excitation 488, emission 507), mNG has a yellow-shifted excitation maximum of 506 nm and an emission maximum of 517 nm, which is more similar to the yellow fluorescent protein EYFP (excitation 514 nm and emission 527). Shaner et al. (2013) recommended to image mNG using standard green fluorescent protein band-pass or long-pass filter sets, or with yellow fluorescent protein filter sets having only minimal reduction in collection efficiency. Therefore, we tested in addition to eGFP the eYFP filter set for mNG fluorescent microscopy. In comparison to the standard eGFP filter set, the usage of the eYFP filter lead to no obvious increase in fluorescence or brightness, only the contrast seems to be increased (Additional file 1: Figure S3). Furthermore, mNG revealed no fluorescence signal with the DsRed filter set (Additional file 1: Figure S3). Therefore, co-localization studies with red fluorescent proteins such as DsRed, mRFP, TagRFP-T, and tdTomato might be applicable in combination with mNG.

**Fig. 5:**
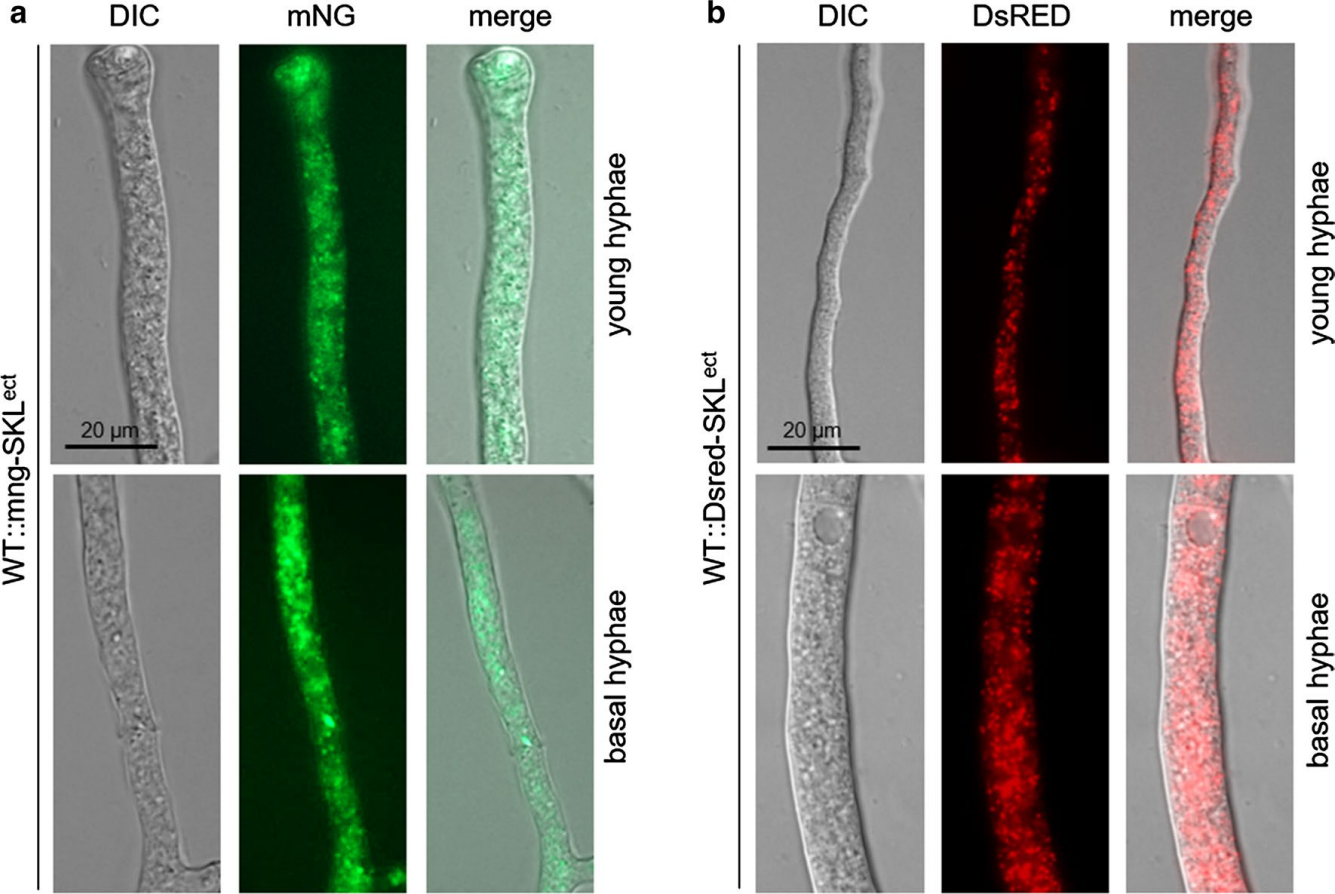
Fluorescence microscopy of peroxisomal-targeted mNG and DsRed in *S. macrospora*. **a** Hyphae of a strain transformed with pmng-SKL. **b** Hyphae of a strain transformed with pDsRed-SKL. The exposure time for image acquisition of both strains was set to be the same. Scale bar, 20 µm

To demonstrate that mNG is suitable to label fungal peroxisomes, we fused the peroxisomal PTS1 signal SKL to the C-terminus of mNG and transformed plasmid pmng-SKL into the *S. macrospora* wild-type strain. Fluorescence microscopy of transformants carrying pmng-SKL led to a punctate fluorescent pattern (Fig. 5a). Similarly, a SKL-tagged DsRed previously used for peroxisomal studies in *S. macrospora* exhibited a punctate peroxisomal fluorescence signal (Fig. 5b) (Elleuche and Pöggeler 2008).

## Discussion

Important innovations in fluorescent protein performance have been described with the discovery of brighter, more readily maturing and more photostable fluorescent proteins (Botman et al. 2019; Heppert et al. 2016). However, often these new proteins have not yet been tested with regard to their potential in filamentous fungi. Here, we expressed the yellow-green fluorescent protein mNG for the first time in *S. macrospora*. By using an inducible and a constitutive promoter, we generated transformants emitting bright and stable green fluorescence. Furthermore, we accomplished visualization of the peroxisomes by tagging mNG with a peroxisomal signal peptide.

The yellow-green fluorescent protein mNG was predicted to be superior to eGFP with regard to its brightness and photostability (Shaner et al. 2013), however, these effects were not that evident when the *mng* gene was expressed in *S. macrospora. *In vivo*,* mNG performed very much alike to eGFP. A similar observation has been described in the nematode *Caenorhabditis elegans* where both fluorescent proteins behaved similarly in most experiments and mNG was only a better tag than eGFP in specific kinds of experiments (Heppert et al. 2016). In *Dictyostelium discoideum* mNG showed an increased brightness in comparison to eGFP (Paschke et al. 2018). Similarly, when using the EYFP filter set instead of the EGFP filter set, the fluorescence of mNG appeared brighter in *S. macrospora*. Therefore, one has to ensure to adapt filter set to mNG fluorescence.

In the yeast *S. cerevisiae*, a recent comprehensive in vivo characterization of 27 fluorescent proteins also revealed that in vitro properties of fluorescent proteins often only poorly reflect their in vivo performance. However, in yeast mNG was among the most photostable fluorescent proteins (Botman et al. 2019). Moreover, in *Nicotiana benthamiana* and *S. cerevisiae,* mNG was shown to be one of the best performing green fluorescent proteins under dynamic pH conditions (Botman et al. 2019; Stoddard and Rolland 2019). These conditions were not tested in our study.

For *S. macrospora,* we used a human codon usage optimized version of *mng*, confirming our previous experience with the expression of a human codon-optimized version of *egfp* (Pöggeler et al. 2003). It is quite possible that a specific *S. macrospora* codon-optimized version of *mng* and/or the addition of synthetic introns will perform even better, as it was shown for modified *mng* versions in human, *C. elegans* and *S. cerevisiae* (Botman et al. 2019; Heppert et al. 2016; Tanida-Miyake et al. 2018).

The selection of a suitable promoter is critical for the expression of heterologous fluorescent proteins in filamentous fungi. We have chosen the constitutive *A. nidulans gpd* promoter successfully applied in a variety of fungal species and additionally the inducible *S. macrospora Smxyl* promoter, previously reported to be functional in *Acremonium chrysogenum* (Bloemendal et al. 2014; Zhang et al. 2020). Therefore, the expression plasmids presented here are likely useful for the expression of *mng* in filamentous fungi other than *S. macrospora*.

Labelling of peroxisomes with reporter fluorescent proteins carrying signal peptides for peroxisomal import is a powerful method for investigating peroxisome dynamics by means of live cell imaging. We showed that mNG-SKL efficiently labels peroxisomes in *S. macrospora*, similar to other fluorescent proteins like eGFP and DsRed carrying a C-terminal SKL tripeptide for peroxisomal matrix import (Elleuche and Pöggeler 2008; Idnurm et al. 2007; Ruprich-Robert et al. 2002).

In conclusion, the vectors for expression of *mng* and labelling of peroxisomes presented here will provide an alternative for tagging of proteins for fungal cell biology and biochemistry. Although the plasmids were primarily constructed for *S. macrospora* they should prove very effective in a wide variety of filamentous as well.

## Supplementary Information

**Additional file 1: Table S1.** Overview of strains used and constructed in this study. **Figure S1.** Lalign (https://embnet.vital-it.ch/software/LALIGN_form.html) alignment of the human codon-optimized mNG version (mNGhco) and the mNG sequence published by (Shaner et al. 2013). **Figure S2.** Western-blot analysis with a NeonGreen antibody of the total protein extracts from a transformant carrying plasmid pGG-C-F-mng to demonstrate *mng* expression under control of the *gpd* promoter. **Figure S3.** Fluorescence microscopy of cytosolic-targeted mNG in *S. macrospora* wt strain under control of the constitutive *gpd* promoter of *A. nidulans*. Fluorescence was recorded with different filter sets: eGFP (Chroma filter set 49002) eYFP (Chroma filter set 49003), DsRed (Chroma filter set 49005). The exposure time for image acquisition of all strains was set to be the same. Scale bar, 50 µm.

## Data Availability

All vectors described are available on request.
