# Supplementary material for: Establishment of the monomeric yellow-green fluorescent protein mNeonGreen for life cell imaging in mycelial fungi
Source: AMB Express. 2020 Dec 21;10:222. doi: 10.1186/s13568-020-01160-x (PMC7752937; doi:10.1186/s13568-020-01160-x)
Supplement: Supplementary file 1 — Additional file 1: Table S1. Overview of strains used and constructed in this study. Figure S1. Lalign (https://embnet.vital-it.ch/software/LALIGN_form.html) alignment of the human codon-optimized mNG version (mNGhco) and the mNG sequence published by (Shaner et al. 2013). Figure S2. Western-blot analysis with a NeonGreen antibody of the total protein extracts from a transformant carrying plasmid pGG-C-F-mng to demonstrate mng expression under control of the gpd promoter. Figure S3. Fluorescence microscopy of cytosolic-targeted mNG in S. macrospora wt strain under control of the constitutive gpd promoter of A. nidulans. Fluorescence was recorded with different filter sets: eGFP (Chroma filter set 49002) eYFP (Chroma filter set 49003), DsRed (Chroma filter set 49005). The exposure time for image acquisition of all strains was set to be the same. Scale bar, 50 µm. [file 13568_2020_1160_MOESM1_ESM.pdf]

Werner et al.

Establishment of the monomeric yellow-green fluorescent protein mNeonGreen for life cell imaging in mycelial fungi

Supplementary material

Table S1: Overview of strains used and constructed in this study.

Figure S1: Lalign (https://embnet.vital-it.ch/software/LALIGN_form.html) alignment of the human codon-optimized mNG version (mNGhco) and the mNG sequence published by (Shaner et al., 2013).

Figure S2: Western-blot analysis with a NeonGreen antibody of the total protein extracts from a transformant carrying plasmid pGG-C-F-mng to demonstrate *mng* expression under control of the *gpd* promoter.

Figure S3: Fluorescence microscopy of cytosolic-targeted mNG in *S. macrospora* wt strain under control of the constitutive *gpd* promoter of *A. nidulans*. Fluorescence was recorded with different filter sets: eGFP (Chroma filter set 49002) eYFP (Chroma filter set 49003 , Chroma filter set 49005 The exposure time for image acquisition of all strains was set to be the same. Scale bar, 50 µm.

Table S1: Overview of strains used and constructed in this study

Strain	Genotype	Source
Escherichia coli		
MACH1	*ΔrecA1398, endA1, tonA, Φ80ΔlacM15, ΔlacX74, hsdR,* (rK- mK+)	Invitrogen
Saccharomyces cerevisiae		
PJ69-4A	*MATa, trp1-901, leu2-3,112, ura3-52, his3-200, ga14Δ, ga18OΔ LYS2::GALl-HIS3, GAL2-ADE2, met2::GAL7-lacZ*	(James et al., 1996)
Sordaria macrospora		
DSM 997	WT	DSMZ
S23442	mutation in *fus1-1* gene, brownish ascospores	(Nowrousian et al., 2012)
WT::xyl-mng^{ect}	ectopic integration of pxyl-mng into DSM997; *nat*^{R}, ssi, fertile, *Pxyl::mng::TtrpC*	This study
WT::gpd-mng^{ect}	ectopic integration of pGG-C-F-mng into DSM997; *nat*^{R}, ssi, fertile, *Pgpd::mng::3xFlag::TtrpC*	This study
WT::1783-1^{ect}	ectopic integration of p1783-1 into DSM997; *hyg*^{R}, ssi, fertile, *Pccg1::egfp::TtrpC*	(Voigt and Pöggeler, 2013)
WT::RHN1^{ect}	ectopic integration of pRHN1 into DSM997; *nat*^{R}, ssi, fertile, *Pccg1::Dsred::TtrpC*	(Werner et al., 2019)
WT::Dsred-SKL^{ect}	ectopic integration of pDsred-SKL into DSM997; *nat*^{R}, ssi, fertile, *Pgpd::Dsred-SKL::TtrpC*	(Elleuche and Pöggeler, 2008)

WT::mng-SKL^{ect}	ectopic integration of pmng-SKL into DSM997; *nat*^{R}, ssi, fertile, *Pgpd::mng-SKL::TtrpC*	This Study

nat^{R} : nourseothricin resistant, *hyg*^{R}: hygromycin resistant; *Pccg1:* promoter of the *clock controlled gene 1* of *Neurospora crassa; Pgpd:* promoter of the glycerinaldehyd-3-phosphat-dehydrogenase-gene of *Aspergillus nidulans; TtrpC:* terminator of the anthranilat synthase gene of *Aspergillus nidulans*; ssi: single spore isolate; SKL: peroxisomal targeting sequence Ser-Arg-Leu; *mng*: gene for green fluorescence protein monomeric NeonGreen, (mNG) of *Branchiostoma lanceolatum*; *Dsred*: gene for red fluorescence protein (DsRed) of *Discosoma* species; *egfp*: gene for green fluorescence protein enhanced green fluorescent protein (eGFP) of *Aequorea victoria*.

```
mNGhco ATGGTGAGCAAGGGCGAGGAGGATAACATGGCCTCTCTGCCAGCCACACATGAGCTGCAC
       :::::::::::::::::::::::::::::::::::::: ::::: ::::::::: : :::
mNG    ATGGTGAGCAAGGGCGAGGAGGATAACATGGCCTCTCTCCCAGCGACACATGAGTTACAC
               10        20        30        40        50        60

               70        80        90       100       110       120
mNGhco ATCTTTGGCTCCATCAACGGCGTGGACTTTGACATGGTGGGCCAGGGCACCGGCAATCCA
       :::::::::::::::::::: :::::::::::::::::::: ::::::::::::::::::
mNG    ATCTTTGGCTCCATCAACGGTGTGGACTTTGACATGGTGGGTCAGGGCACCGGCAATCCA
               70        80        90       100       110       120

              130       140       150       160       170       180
mNGhco AATGATGGCTATGAGGAGCTGAACCTGAAGTCCACCAAGGGCGACCTACAGTTCTCCCCC
       :::::::: ::::::::: : :::::::::::::::::::: ::::: ::::::::::::
mNG    AATGATGGTTATGAGGAGTTAAACCTGAAGTCCACCAAGGGTGACCTCCAGTTCTCCCCC
              130       140       150       160       170       180

              190       200       210       220       230       240
mNGhco TGGATTCTGGTCCCTCATATCGGGTATGGCTTCCATCAGTACCTGCCCTACCCTGACGGG
       ::::::::::::::::::::::::::::::::::::::::::::::::::::::::::::
mNG    TGGATTCTGGTCCCTCATATCGGGTATGGCTTCCATCAGTACCTGCCCTACCCTGACGGG
              190       200       210       220       230       240

              250       260       270       280       290       300
mNGhco ATGAGCCCTTTCCAGGCCGCCATGGTGGATGGCTCTGGATACCAGGTCCATCGCACAATG
       :::   :::::::::::::::::::: :::::::: :::::::: :::::::::::::::
mNG    ATGTCGCCTTTCCAGGCCGCCATGGTAGATGGCTCCGGATACCAAGTCCATCGCACAATG
              250       260       270       280       290       300

              310       320       330       340       350       360
mNGhco CAGTTTGAAGATGGCGCCTCCCTGACTGTGAACTACCGCTACACCTACGAGGGAAGCCAC
       :::::::::::::: :::::::: ::::: ::::::::::::::::::::::::::::::
mNG    CAGTTTGAAGATGGTGCCTCCCTTACTGTTAACTACCGCTACACCTACGAGGGAAGCCAC
              310       320       330       340       350       360

              370       380       390       400       410       420
mNGhco ATCAAAGGAGAGGCCCAGGTGAAGGGGACTGGCTTCCCTGCTGACGGCCCTGTGATGACC
       :::::::::::::::::::::::::::::::: :::::::::::::: ::::::::::::
mNG    ATCAAAGGAGAGGCCCAGGTGAAGGGGACTGGTTTCCCTGCTGACGGTCCTGTGATGACC
              370       380       390       400       410       420

              430       440       450       460       470       480
mNGhco AACAGCCTGACCGCTGCCGACTGGTGCAGGAGCAAGAAGACTTACCCCAACGACAAAACC
       :::   ::::::::::: ::::::::::::   :::::::::::::::::::::::::::
mNG    AACTCGCTGACCGCTGCGGACTGGTGCAGGTCGAAGAAGACTTACCCCAACGACAAAACC
              430       440       450       460       470       480

              490       500       510       520       530       540
mNGhco ATCATCAGTACCTTTAAGTGGAGTTACACCACTGGAAATGGCAAACGCTACCGGAGCACT
       :::::::::::::::::::::::::::::::::::::::::::: :::::::::::::::
mNG    ATCATCAGTACCTTTAAGTGGAGTTACACCACTGGAAATGGCAAGCGCTACCGGAGCACT
              490       500       510       520       530       540

              550       560       570       580       590       600
mNGhco GCCCGGACCACCTACACCTTTGCCAAGCCAATGGCCGCTAACTATCTGAAGAACCAGCCC
       :: :::::::::::::::::::::::::::::::: ::::::::::::::::::::::: 
mNG    GCGCGGACCACCTACACCTTTGCCAAGCCAATGGCGGCTAACTATCTGAAGAACCAGCCG
```

```
                550       560       570       580       590       600

                610       620       630       640       650       660
mNGhco ATGTACGTGTTCCGAAAGACCGAGCTGAAGCACTCCAAGACCGAGCTGAACTTCAAGGAG
       :::::::::::::: ::::: ::::: ::::::::::::::::::: :::::::::::
mNG    ATGTACGTGTTCCGTAAGACGGAGCTCAAGCACTCCAAGACCGAGCTCAACTTCAAGGAG
                610       620       630       640       650       660

                670       680       690       700       710
mNGhco TGGCAGAAGGCCTTTACCGATGTGATGGGCATGGACGAGCTGTACAAGTAG
       ::::: ::::::::::::::::::::::::::::::::::::::::::::
mNG    TGGCAAAAGGCCTTTACCGATGTGATGGGCATGGACGAGCTGTACAAGTAA
                670       680       690       700       710
```

Supplementary Figure 1 Lalign (https://embnet.vital-it.ch/software/LALIGN_form.html) alignment of the human codon-optimized mNG version (mNGhco) and the mNG sequence published by (Shaner et al., 2013). Changes of the sequence are framed in grey.

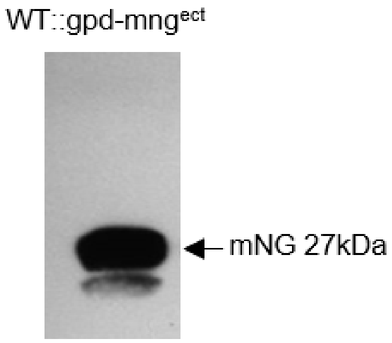

Figure S2: Western-blot analysis with a NeonGreen antibody of the total protein extracts from a transformant carrying plasmid pGG-C-F-mng to demonstrate *mng* expression under control of the *gpd* promoter.

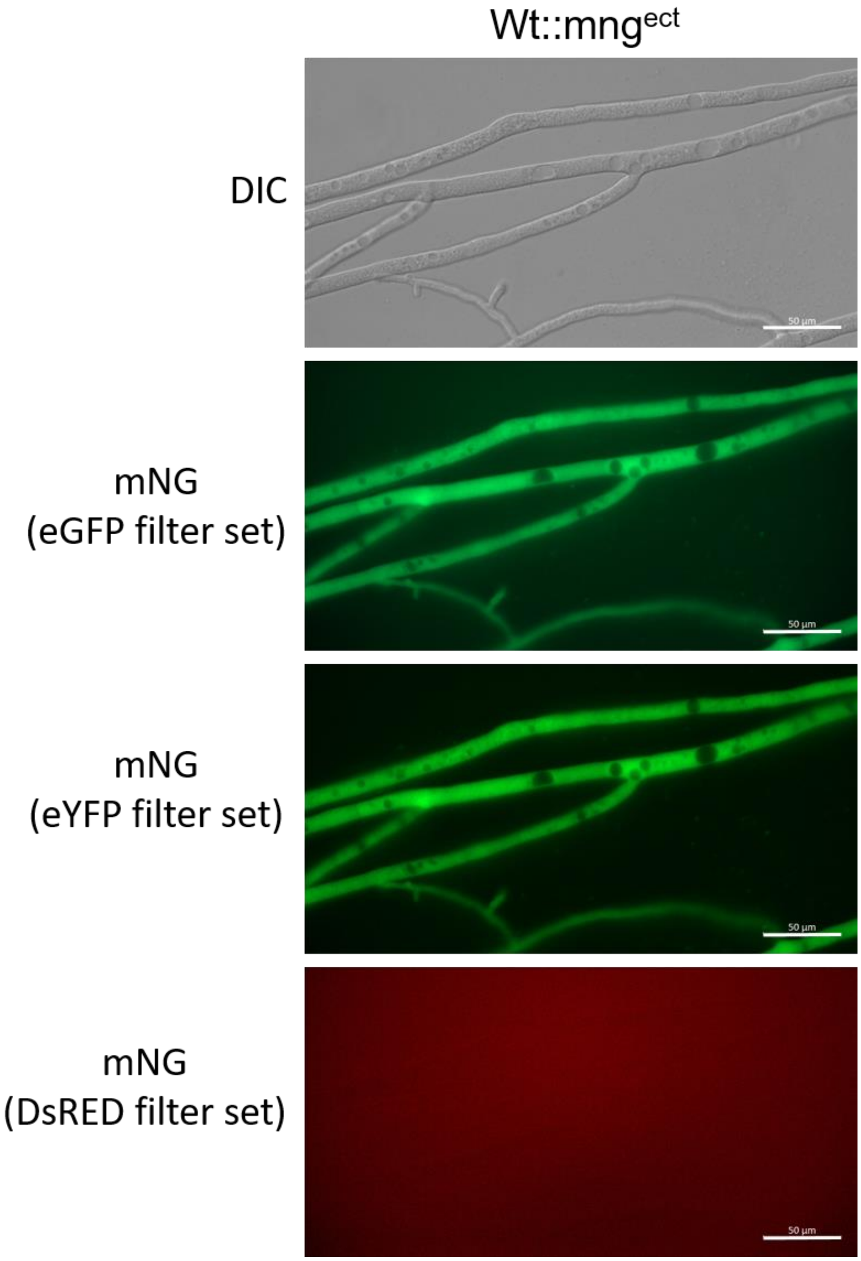

Figure S3: Fluorescence microscopy of cytosolic-targeted mNG in *S. macrospora* wt strain. *mng* is under control of the constitutive *gpd* promoter of *A. nidulans*. Fluorescence was recorded with different filter sets: eGFP (Chroma filter set 49002), eYFP (Chroma filter set 49003), DsRed (Chroma filter set 49005). The exposure time for image acquisition of all strains was set to be the same. Scale bar, 50 µm.

References

Elleuche S, Pöggeler S (2008) Visualization of peroxisomes via SKL-tagged DsRed protein in *Sordaria macrospora*. Fungal Genet Rep 55**:**8-12

James P, Halladay J, Craig EA (1996) Genomic libraries and a host strain designed for highly efficient two-hybrid selection in yeast. Genetics 144**:**1425-1436

Nowrousian M, Teichert I, Masloff S, Kück U (2012) Whole-genome sequencing of *Sordaria macrospora* mutants identifies developmental genes. G3 (Bethesda) 2**:**261-270

Shaner NC, Lambert GG, Chammas A, Ni Y, Cranfill PJ, Baird MA, Sell BR, Allen JR, Day RN, Israelsson M, Davidson MW, Wang J (2013) A bright monomeric green fluorescent protein derived from *Branchiostoma lanceolatum*. Nat Methods 10**:**407-409

Voigt O, Pöggeler S (2013) Autophagy genes *Smatg8* and *Smatg4* are required for fruiting-body development, vegetative growth and ascospore germination in the filamentous ascomycete *Sordaria macrospora*. Autophagy 9**:**33-49

Werner A, Herzog B, Voigt O, Valerius O, Braus GH, Pöggeler S (2019) NBR1 is involved in selective pexophagy in filamentous ascomycetes and can be functionally replaced by a tagged version of its human homolog. Autophagy 15**:**78-97
